# A rare systemic etiology of heart failure and liver dysfunction

**DOI:** 10.1002/ccr3.2229

**Published:** 2019-06-03

**Authors:** Christopher Lee, Edward Chau, Sumit Patel, Diana Zhao, Alexander H. Yang, Brian T. Lee, Jacob Alexander, Enrique Ostrzega, Patrick Sarte

**Affiliations:** ^1^ University of Southern California Los Angeles California; ^2^ Internal Medicine Keck School of Medicine of the University of Southern California Los Angeles California

**Keywords:** amyloidosis, hepatic dysfunction, infiltrative cardiomyopathy, restrictive cardiomyopathy

## Abstract

Systemic amyloidosis is a rare condition that can manifest with cardiomyopathy, hepatic dysfunction, and renal disease. Diagnosis is often missed and/or delayed due to chronic multi‐system involvement and indeterminate signs and symptoms. Treatment generally involves systemic therapy and autologous stem‐cell transplantation.

## 1 INTRODUCTION

Systemic amyloidosis is a rare etiology of infiltrative cardiomyopathy associated with hepatic dysfunction, renal disease, and peripheral neuropathy.^1^ We report a case of immunoglobulin light chain (AL) amyloidosis causing symptomatic cardiomyopathy.

## 2 CASE REPORT

**Figure 1:**
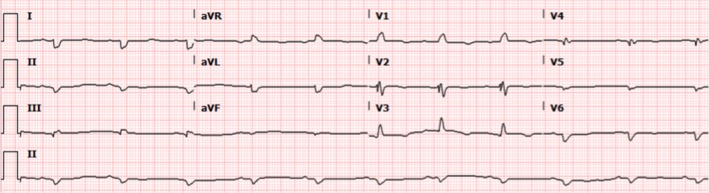
Electrocardiogram showing sinus rhythm with low voltages, a right bundle branch block, left posterior fascicular block, and poor R wave progression

**Figure 2:**
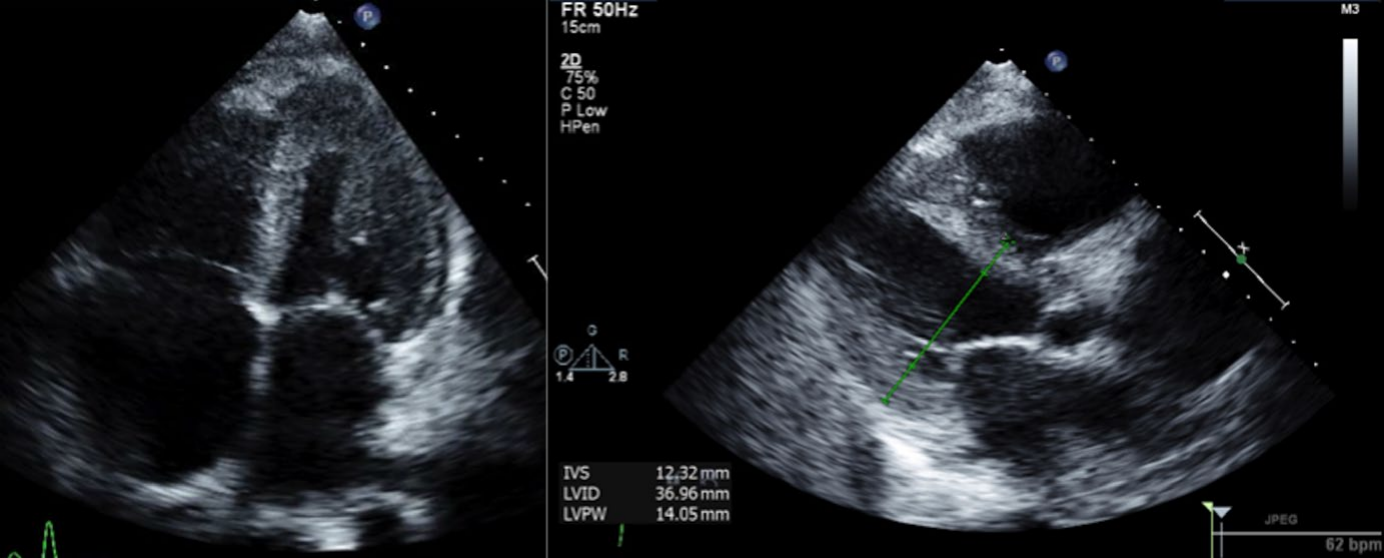
Apical 4 chamber and parasternal long axis echocardiographic views showing left ventricular hypertrophy (posterior and septal wall thickness—14 and 12 mm, respectively) along with severely increased right ventricular and right atrial cavity size

**Figure 3:**
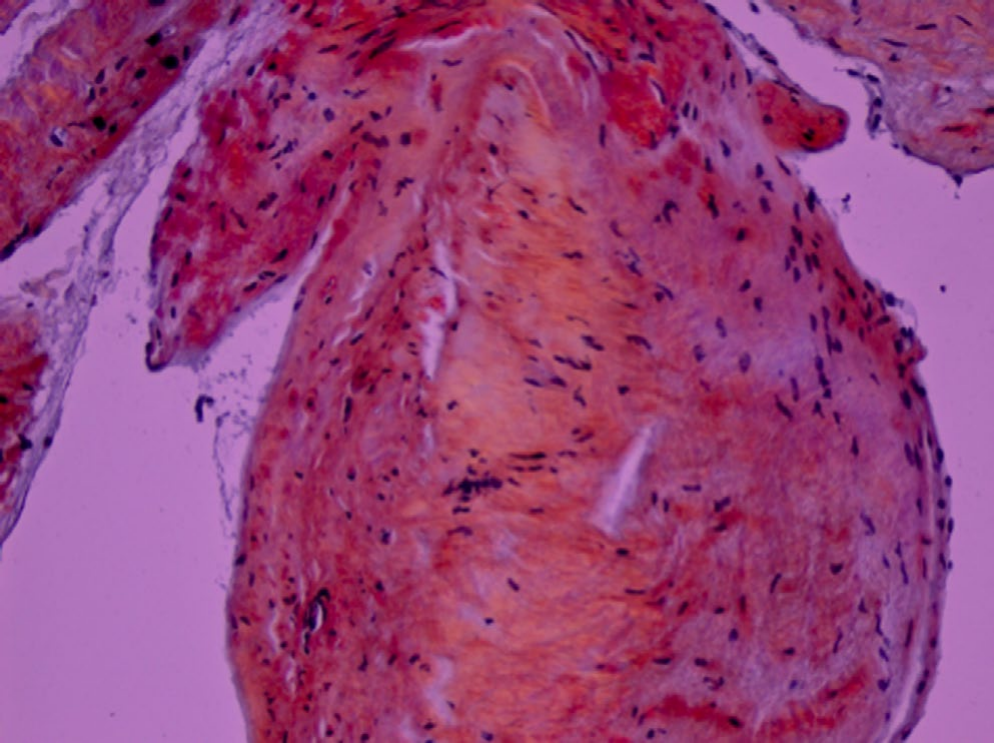
Right ventricular myocardial biopsy with Congo Red staining showing apple‐green birefringence, characteristic of amyloid deposits, under polarized light, 200×

A 65‐year‐old male with diabetes mellitus type 2, hypertension, cryptogenic cirrhosis, and possible heart failure presented with shortness of breath, worsening fatigue and dyspnea on exertion for several months. He noted worsening orthopnea but denied chest pain, palpitations, fevers, chills, night sweats, diarrhea, melena, or hematochezia. Physical exam revealed a nontender but distended abdomen with a fluid wave and hepatomegaly. Venous stasis changes were present in the lower extremities along with 2 mm pitting edema. Electrocardiogram (EKG) showed normal sinus rhythm with low voltages, a right bundle branch block, and left posterior fascicular block (Figure 1). Troponin T was elevated to 0.13 ng/mL (<0.01 ng/mL) but remained stable at 0.11 ng/mL. Probrain natriuretic peptide (proBNP) was elevated to 8870 pg/mL (<125 pg/mL). Serum chemistries were only notable for a creatinine of 1.71 mg/dL (0.5‐1.3 mg/dL), albumin 2.8 g/dL (3.5‐5.0 g/dL), total protein 7.2 g/dL (6.0‐8.0 g/dL), alkaline phosphatase 341 U/L (40‐129 U/L), AST 24 U/L (10‐40 U/L), and ALT 15 U/L (10‐55 U/L). Serum protein electrophoresis was completed and revealed a homogeneous band in the beta region (2.4 g/dL) identified by immunofixation as IgG/Lambda. Urine protein electrophoresis showed the same IgG/Lambda band. Serum light chains were elevated—kappa light chains 63.1 mg/L (3.3‐19.4 mg/L) and lambda light chains 287.1 mg/L (5.7‐26.3 mg/L). Abdominal ultrasound showed a coarsened and nodular liver. Transthoracic echocardiogram revealed a left ventricular ejection fraction of 35% with diffuse hypokinesis, wide‐open tricuspid regurgitation, left ventricular posterior wall (LVPW) thickness 14 mm (6‐10 mm), and interventricular septal thickness 12 mm (6‐10 mm) (Figure 2). Coronary angiogram was without evidence of coronary artery disease. Fat pad, liver, and cardiac biopsies were obtained, all of which showed apple‐green birefringence under polarized light (Figure 3). Electron microscopy confirmed the diagnosis of AL amyloidosis. The patient was started on cyclophosphamide‐bortezomib‐dexamethasone (CyBorD) but eventually succumbed to his disease several months later.

## 3 DISCUSSION

Immunoglobulin light chain amyloidosis is a rare etiology of infiltrative cardiomyopathy due to amyloid protein deposition.^1^ Recent epidemiological findings in the United States from 2007 to 2015 found the incidence of AL amyloidosis to range from 10.8 to 15.2 cases per million person‐years.^2^ Given chronic multi‐system involvement, symptoms are generally nonspecific, making diagnosis challenging. Hepatic involvement may occur presenting as hepatomegaly, usually with a characteristically elevated alkaline phosphatase and only mildly elevated transaminases due to sinusoidal infiltration. Renal involvement may also occur presenting chiefly as proteinuria and renal failure.^3^

In 50% of cases, amyloid fibril deposition may affect the cardiac system as well.^1^, ^4^ Cardiac amyloidosis usually manifests as a restrictive cardiomyopathy and eventual systolic biventricular failure. Initial symptoms may include progressive dyspnea on exertion, peripheral edema, and at times, exertional or postprandial syncope.^5^ Troponins may be persistently elevated and may be secondary to persistent myocyte ischemia and necrosis due to decreased myocardial flow reserve. EKG findings include low voltages, bundle branch blocks, and arrhythmias such as supraventricular tachycardias (eg, atrial fibrillation) and ventricular tachycardia.^6^ Echocardiographic findings include concentric left and right ventricular wall thickening, a restrictive filling pattern, atrial septal infiltration, and eventual progressive systolic dysfunction. Other imaging modalities, such as cardiac magnetic resonance imaging, help to further quantify morphologic data of myocardial tissue, further suggesting the diagnosis of cardiac amyloidosis. Although these constellations of findings and multi‐system organ involvement may suggest a diagnosis of AL amyloidosis, tissue biopsy is ultimately required. Cardiac amyloidosis may be diagnosed either with histologic confirmation of amyloid deposits on an endomyocardial biopsy or echocardiographic findings suggestive of cardiac amyloidosis in a patient with an established noncardiac biopsy positive for amyloidosis.^4^

Once the diagnosis has been established, treatment generally involves systemic therapy and eventual autologous stem‐cell transplantation (ASCT). CyBorD is now one of the most commonly utilized initial chemotherapy regimens in AL amyloidosis.^7^ Goal‐directed heart failure treatment may be initiated; however, beta‐blockers, angiotensin‐converting enzyme inhibitors (ACE inhibitors), and angiotensin receptor blockers (ARBs) should be utilized with caution. Beta‐blockers may be poorly tolerated in patients with restrictive cardiomyopathy due to worsened cardiac output while renal function may be a concern with the use of ACE inhibitors and ARBs.^5^ The role of device therapy with implantable cardioverter defibrillators (ICDs) is also uncertain, as limited data has not shown a survival benefit in patients with amyloid cardiomyopathy.^8^

Although uncommon, clinicians should be vigilant of these symptoms and include systemic amyloidosis within the differential diagnosis when evaluating patients with cardiomyopathy.

## CONFLICT OF INTEREST

None declared.

## AUTHOR CONTRIBUTION

CL: wrote manuscript, revised manuscript, and figure acquisition. EC: revised manuscript. SP: revised manuscript and figure acquisition. DZ: revised manuscript. AHY: revised manuscript. BTL: revised manuscript. JA: revised manuscript. EO: revised manuscript. PS: revised manuscript.
